# Advances in *Pseudomonas aeruginosa*-Induced Programmed Cell Death and Potential Targeted Treatment Strategies

**DOI:** 10.3390/microorganisms13112560

**Published:** 2025-11-10

**Authors:** Chunjiang Tan, Yifeng Luo

**Affiliations:** 1 Division of Pulmonary and Critical Care Medicine, The First Affiliated Hospital of Sun Yat-sen University, Guangzhou 510080, China; 2 Institute of Respiratory Diseases, Sun Yat-sen University, Guangzhou 510080, China; 3 Department of Emergency Medicine, The First Affiliated Hospital of Sun Yat-sen University, Guangzhou 510080, China

**Keywords:** *P. aeruginosa*, programmed cell death, PANoptosis, apoptosis, pyroptosis, necroptosis, ferroptosis

## Abstract

*Pseudomonas aeruginosa* (*P. aeruginosa*) is responsible for the high prevalence of various nosocomial infections, and it is challenging to completely eradicate *P. aeruginosa* infection in clinics. One of *P. aeruginosa*’s main pathogenic mechanisms is to trigger multiple forms of programmed cell death (PCD), such as apoptosis, pyroptosis, necroptosis, ferroptosis, and PANoptosis, among which PANoptosis is a newly discovered PCD pathway mediated by PANoptosome complexes and their key upstream regulators. Compared with other well-studied PCD pathways, advances in *P. aeruginosa*-induced PANoptosis have yet to be thoroughly reviewed. This review highlights research advances in this pathway, providing mechanistic insights by summarizing the upregulation and/or activation of PANoptosome sensor proteins and their key upstream regulators during *P. aeruginosa* infection. We also offer perspectives on the mechanistic links between *P. aeruginosa* cytotoxicity and other forms of PCD. Additionally, pharmacological compounds that may be used to target *P. aeruginosa*-induced PCD, particularly PANoptosis, are discussed, and future research and therapeutic directions are proposed. Our work helps bridge the knowledge gap, paving the way for further understanding of *P. aeruginosa*-induced PCD and the development of novel therapeutics against *P. aeruginosa* infection.

## 1. Introduction

*Pseudomonas aeruginosa* (*P. aeruginosa*) is a highly virulent and versatile pathogen that causes various nosocomial infections [1,2]. Among Gram-negative bacteria, a high prevalence of *P. aeruginosa* has been reported in a wide range of nosocomial infections such as pneumonia [1,3], bloodstream infections [4,5], urinary tract infection [6], intra-abdominal infections [7], and sepsis [8]. Of note, as an opportunistic pathogen, critically ill and immunocompromised patients are more susceptible to *P. aeruginosa*-associated infections [9]. Moreover, drug-resistant strains even contribute to increased disease severity [10]. *P. aeruginosa* is one of the leading bacterial pathogens, accounting for more than 500,000 deaths in 2019, with a high age-standardized mortality rate of 7.4% [11]. Although new-generation antibiotics have been put into clinical use, it still remains challenging to completely eradicate *P. aeruginosa* infection due to its high level of antibiotic resistance [12,13]. To explore alternative and effective therapeutics against this infection, it is crucial to further investigate the pathogenicity of *P. aeruginosa*.

One of the main pathogenic mechanisms of *P. aeruginosa* is to induce multiple forms of cell death [14]. Previously published research has demonstrated the cytotoxicity of *P. aeruginosa* since multiple forms of programmed cell death (PCD) can be induced by *P. aeruginosa* virulence factors. For example, exotoxins secreted from bacterial type III secretion system (T3SS) cause cell apoptosis [15]. The lipopolysaccharide (LPS) of *P. aeruginosa* is responsible for triggering cell pyroptosis [16]. Furthermore, *P. aeruginosa* exolysin leads to the activation of necroptosis [17]. It is noteworthy that PANoptosis, a new form of PCD that shares key molecular characteristics of apoptosis, pyroptosis, and necroptosis, has been demonstrated to occur in various infectious diseases [18,19,20]. Pathogen-induced PANoptosis (e.g., by *SARS-CoV-2* or *F. novicida*) has been shown to drive cell death and disease pathology [19,20], and, importantly, preventing PANoptosis significantly reduces mortality in preclinical models of infection [19]. Crucially, works from Kanneganti’s team [21] and Liu and colleagues [22] provided evidence that *P. aeruginosa* triggers PANoptosis in target cells. As a Gram-negative opportunistic pathogen similar to *F. novicida*, *P. aeruginosa*-induced PANoptosis is proposed to contribute to disease progression. However, compared with other well-studied PCD pathways (e.g., apoptosis and pyroptosis), advances in *P. aeruginosa*-induced PANoptosis have yet to be comprehensively reviewed. In addition, progress in other forms of *P. aeruginosa*-induced PCD needs to be examined.

In this review, we focus on providing up-to-date, integrated, and critical perspectives on the close association between *P. aeruginosa* cytotoxicity and the induction of multiple PCD pathways. Moreover, potential treatment strategies targeting *P. aeruginosa*-induced PCD, particularly PANoptosis, are discussed. Gaining new insights into *P. aeruginosa*-induced PCD may enhance the understanding of *P. aeruginosa*’s pathogenicity, thereby boosting the discovery of novel therapeutics against *P. aeruginosa* infection.

## 2. *P. aeruginosa*-Induced PCD

### 2.1. P. aeruginosa-Induced PANoptosis

**Figure 1:**
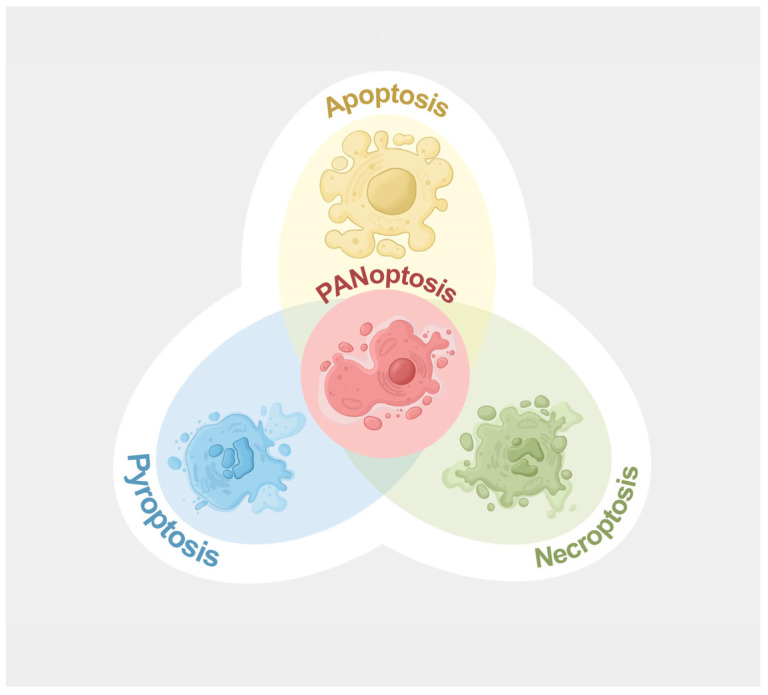
A simplified illustration of the interplay among PANoptosis, apoptosis, pyroptosis, and necroptosis. Figure created using Procreate v5.4.7.

As described in the Introduction, *P. aeruginosa* is able to induce multiple forms of PCD such as apoptosis [15], pyroptosis [16], and necroptosis [23], which are essential biological processes that regulate immune response to pathogen invasion. As these PCD pathways are collectively involved in host immune defense, potential cross-talk may exist among them. In 2019, a new form of PCD that shares important molecular features of apoptosis, pyroptosis, and necroptosis was discovered and termed PANoptosis [24], highlighting the intricate interplay among these PCD pathways (Figure 1). It is critical to point out that individual inhibition of key molecular components of apoptosis, pyroptosis, or necroptosis cannot prevent PANoptosis, and only the combined deletion of PANoptotic components can effectively abrogate the PANoptosis process. PANoptosis has been reported to occur in various infectious diseases [20,25,26], including Gram-negative bacteria infection [18,20], and is regulated by the PANoptosome complexes and their key upstream regulators, as discussed below.

#### 2.1.1. Composition of PANoptosome Complexes

The process of PANoptosis is sophisticatedly regulated by the PANoptosome, a multiprotein complex comprising pivotal molecular components of apoptosis, pyroptosis, and necroptosis [25]. Although multiple signal pathways are involved in the formation of PANoptosome complexes and the composition of each complex may vary under different stimuli [20,24,25], the PANoptosome is primarily composed of the following proteins.

Sensor proteins: (1) Z-DNA binding protein 1 (ZBP-1). ZBP-1 is heavily engaged in innate immunity and cell death through the sensing of aberrant nucleic acids [27,28]. Notably, apart from nucleic acid recognition, ZBP-1 functions as an essential pathogen sensor to regulate cell death and inflammatory responses [29]. Activated ZBP-1 promotes the assembly of the PANoptosome by mediating the recruitment and interaction of other molecular components of PANoptosis, such as caspase-8 and receptor-interacting serine/threonine-protein kinase 3 (RIPK3), in the context of Gram-negative bacteria infection [24,25]. Importantly, ZBP-1 deficiency or mutation leads to attenuated levels of PANoptosis [20]. (2) Absent in melanoma 2 (AIM2). This well-known DNA sensor is essential for host immune defense against Gram-negative bacteria infection [30,31]. The work from Lee and colleagues reveals that AIM2 controls PANoptosome formation by cooperating with ZBP-1 and Pyrin [20]. In cells with AIM2 deficiency, PANoptosis is totally abrogated, accompanied by reduced expression levels of ZBP-1 and Pyrin [20]. Furthermore, complete inactivation of PANoptotic molecules (e.g., caspase-8, -3, -7, -1, Gasdermin D, and RIPK3) is found in AIM2-deficient cells [20]. These results suggest the important role of AIM2 in PANoptosis. (3) Receptor-interacting serine/threonine-protein kinase 1 (RIPK1). In addition to ZBP-1 and AIM2, Gram-negative bacteria infection induces the assembly of the RIPK1-PANoptosome, which is crucial for regulating cell death and host antimicrobial defense [18]. Importantly, loss of RIPK1 leads to a reduction in PANoptotic molecules (e.g., caspase-8, -3, -7, -1, and Gasdermin D), along with decreased levels of pyroptosis and apoptosis [18]. Interestingly, necroptosis is activated upon loss of RIPK1 [18], and it might act as a compensatory mechanism to preserve immune homeostasis in response to bacterial infection. (4) NLR family pyrin domain containing 12 (NLRP12). Recently, the NLRP12-PANoptosome has been discovered upon heme plus pathogen-associated molecular pattern (PAMP) stimulation [32], implicating its regulatory role in infectious and inflammatory diseases.

Adaptor proteins: (1) Apoptosis-associated speck-like protein containing CARD (ASC). This functions as an adaptor that bridges the interaction between upstream signaling and downstream effector proteins [33,34]. The interactions of ASC with molecular components of PANoptosis (e.g., ZBP-1, AIM2, Pyrin, RIPK1, caspase-1, and RIPK3) are observed following infection, and ASC deletion leads to decreased expression of ZBP-1, Pyrin, and caspase-1 [20]. (2) FAS-associated via death domain (FADD). This serves as an adaptor for caspase enzymes (e.g., caspase-8) and is engaged in the regulation of inflammation and cell death [35,36]. Notably, loss of FADD diminishes both the PANoptotic molecule activation and cell death [19].

**Figure 2:**
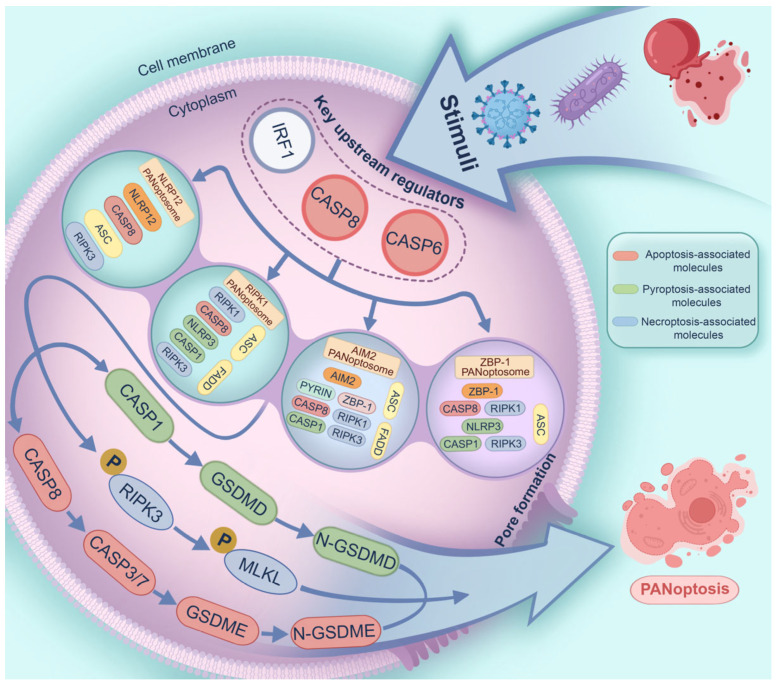
Molecular processes of PANoptosis. Diverse stimuli, such as viruses, bacteria, and heme plus PAMPs, lead to upregulation and/or activation of key upstream regulators of the PANoptosome (e.g., IRF1, caspase-8, and -6). These regulators then mediate the assembly of the ZBP-1 PANoptosome (ZBP-1, RIPK1, caspase-8, NLRP3, RIPK3, caspase-1, and ASC), AIM2 PANoptosome (AIM2, ZBP-1, Pyrin, RIPK1, caspase-8, RIPK3, caspase-1, ASC, and FADD), RIPK1 PANoptosome (RIPK1, caspase-8, NLRP3, caspase-1, RIPK3, ASC, and FADD), or NLRP12 PANoptosome (NLRP12, RIPK3, caspase-8, and ASC). The downstream effectors are subsequently activated, thereby contributing to plasma membrane pore formation and PANoptosis. Apoptosis-, pyroptosis-, and necroptosis-associated molecules are shown in red, green, and blue, respectively.

Effector proteins: (1) Caspase-1. This is a crucial effector of PCD, and it can specifically cleave Gasdermin D (GSDMD) to form pores in the plasma membrane [37]. (2) RIPK3. This phosphorylates downstream protein mixed-lineage kinase domain-like protein (MLKL), and activated MLKL traffics to the cell membrane for pore formation [38]. In the context of PANoptosis, RIPK3 is co-expressed with other molecular components of PANoptosis, such as ZBP1, RIPK1, and ASC [25], suggesting that these proteins assemble into the PANoptosome complex. (3) Caspase-8. This is a PANoptotic caspase that mediates the cleavage and activation of downstream caspase-3 and -7, as well as GSDMD, Gasdermin E (GSDME), and RIPK3 [39]. Active caspase-3 and -7 drive membrane pore generation via the cleavage of GSDME [39,40]. Together, these effector proteins jointly contribute to pore formation in the plasma membrane, ultimately driving PANoptosis in a synergistic manner. Figure 2 presents the molecular processes of PANoptosis and four types of PANoptosome complexes, and highlights apoptosis-, pyroptosis-, and necroptosis-associated molecules involved in PANoptosis.

#### 2.1.2. Key Upstream Regulators of PANoptosome Complexes

Although the sensor, adaptor, and effector proteins discussed above together constitute the PANoptosome, it is essential to identify upstream molecules mediating PANoptosome formation. Interferon regulatory factor 1 (IRF1) has been discovered to serve as an upstream regulator in the PANoptosis pathway [19]. IRF1-deficient cells are protected from PANoptosis in response to tumor necrosis factor-α and IFN-γ co-treatment, along with decreased activation of both PANoptotic molecules (e.g., caspase-3, -7, and -8) and pore-forming molecules (e.g., GSDME and MLKL) [19]. More importantly, prior studies have confirmed the key role of IRF1 in regulating the expression of PANoptosome sensor proteins. For example, IRF1 was identified as a transcriptional modulator of ZBP1 because the level of ZBP1 is diminished in cells with IRF1 deficiency during infection [41]. IRF1 has also been confirmed to contribute to the formation and activation of ZBP1-PANoptosome [42]. Similar to ZBP-1, results from another study show that both AIM2 activation and host survival require IRF1 during *F. novicida* infection [31], a Gram-negative bacterium demonstrated to induce PANoptosis [20]. In addition to these, loss of IRF1 leads to impaired activation of RIPK1 and PANoptotic molecules, as well as reduced RIPK1-mediated PANoptosis [42], revealing that IRF1 promotes the formation of RIPK1-PANoptosome. Recently, the regulatory role of IRF1 in mediating NLRP12 expression and NLRP12-dependent PANoptosis has been recognized, as IRF1-deficient cells have significantly lower NLRP12 expression and cell death [32]. All the evidence mentioned above proves that IRF1 acts as a key upstream regulator for orchestrating PANoptosome assembly and PANoptosis.

Apart from IRF1, growing evidence has indicated the importance of caspase-8 in regulating the emergence of PANoptosis. For instance, work from Karki and colleagues unveils that caspase-8 is a core molecule involved in triggering PANoptosis because cells with ablation of caspase-8 and RIPK3 are completely resistant to PANoptosis [19]. Interestingly, loss of RIPK3 alone does not protect cells against PANoptosis [19]. (Cells from mice with caspase-8 deficiency alone are not available, due to embryonic lethality [43]). Furthermore, substantially declined activation of PANoptosis-associated caspases (e.g., caspase-3 and -7) and downstream pore-forming proteins are found upon deletion of caspase-8 and RIPK3 [19]. Results from another study show that caspase-8 functions as a pivotal molecular switch that intricately controls the process of apoptosis, pyroptosis, and necroptosis [44]. Additionally, caspase-8 was demonstrated to closely interact with AIM2-ASC complex upon *F. novicida* infection [45]. Aside from caspase-8, caspase-6 associates with RIPK3 to facilitate RIPK3-ZBP1 interaction, promoting PANoptosis and innate immunity activation [46]. Activation levels of PANoptosis-associated caspases and downstream effector proteins are attenuated upon caspase-6 deficiency [46]. These findings demonstrate that caspase-8 and -6 act as core upstream regulators of PANoptosome complexes, and they are capable of interacting with PANoptosome components to modulate the process of PANoptosis.

#### 2.1.3. Progress in *P. aeruginosa*-Induced PANoptosis

As a leading Gram-negative pathogen responsible for various nosocomial infections, growing evidence has revealed that the severe cytotoxicity of *P. aeruginosa* drives PANoptosis. A study from Kanneganti’s team first discovered that the *P. aeruginosa* PAO1 strain triggers PANoptosis in mouse bone marrow-derived macrophages, and cells with concomitant loss of caspase-8, RIPK3, and caspase-1/11 are fully protected from PANoptosis [21]. Nevertheless, this research did not include in vivo data, and the potential influences of host microenvironment (e.g., pH and metabolites) and immune cell interactions on PANoptosis should not be overlooked. Another study identified that a low dose of *P. aeruginosa* causes PANoptosis in tumor cells, as evidenced by concurrent activation of apoptotic and necroptotic cell death pathways [47]. Notably, a recently published study shows that the PAO1 strain with rhl (a subsystem of the bacterial quorum sensing (QS) network) deficiency induces PANoptosis in mouse bone marrow-derived macrophages [22]. More importantly, mice are resistant to infection caused by the PAO1 strain with dual deletion of rhl and pqs (another subsystem of QS) [22], demonstrating that *P. aeruginosa*-induced PANoptosis is intricately regulated by the bacterial QS system. All these findings confirm that *P. aeruginosa* infection leads to PANoptosis. However, the underlying mechanisms of *P. aeruginosa*-induced PANoptosis are still largely unknown. Interestingly and importantly, accumulating evidence has demonstrated that *P. aeruginosa* infection promotes the upregulation and/or activation of PANoptosome sensor proteins as well as key upstream regulators of PANoptosome complexes, providing mechanistic evidence for the emergence of PANoptosis in the context of *P. aeruginosa* infection, which are summarized below.

##### Upregulation and/or Activation of PANoptosome Sensor Proteins Upon *P. aeruginosa* Infection

As previously summarized, ZBP-1, AIM2, RIPK1, and NLRP12 are crucial sensor proteins of the PANoptosome. As an important pathogen sensor orchestrating cell death and inflammatory responses, enhanced expression of ZBP-1 is detected in neutrophils following *P. aeruginosa* infection, along with augmented levels of neutrophil cell death [23]. This result implies that *P. aeruginosa* infection is closely associated with potential neutrophil PANoptosis. Furthermore, prior research has shown that ZBP-1 interacts with other components of PANoptosis, including RIPK3, RIPK1, and caspase-8, to intricately regulate cell death and immune response [48]. More importantly, as previously described, ZBP-1 deficiency or mutation attenuates PANoptosis [20]. These findings strongly support the ability of *P. aeruginosa*-induced ZBP-1 expression to trigger PANoptosis because of ZBP-1′s pivotal role in promoting PANoptosome assembly and the interaction between the PANoptosome components.

Results from a prior study show that AIM2 expression (another important PANoptosome sensor protein) is elevated in both in vivo and in vitro models of pulmonary *P. aeruginosa* infection [49]. Considering the key role of AIM2 in PANoptosome formation as discussed earlier, *P. aeruginosa*-induced AIM2 upregulation appears to contribute to the arise of PANoptosis. It is worth noting that inhibition of AIM2 activation alleviates the level of pulmonary inflammation and severity of *P. aeruginosa* pneumonia [49], indicating a close link between AIM2-mediated PANoptosis and the pathogenesis of pulmonary *P. aeruginosa* infection.

It is important to note that works from Sundaram et al. provide evidence that the phosphorylation level of RIPK1 is upregulated in a cell model of *P. aeruginosa* infection, confirming the activation of RIPK1 [21]. Intriguingly, activation of caspase-8 and -1 is also observed in this model [21], both of which are important components of RIPK1-PANoptosome. Moreover, *P. aeruginosa*-induced PANoptosis is totally abrogated in cells with combined deficiency of caspase-8, RIPK3, and caspase-1/11 [21]. Collectively, these results reveal that RIPK1 is strongly engaged in PANoptosome assembly and PANoptosis through interaction with caspase-1, caspase-8, and RIPK3 in the context of *P. aeruginosa* infection.

Regarding NLRP12, it is involved in host defense against *P. aeruginosa* infection as NLRP12-deficient mice are more susceptible to this pathogen [50]. Nevertheless, to date, NLRP12 has only been shown to control PANoptosome formation following heme and PAMP stimulation [32]. Together, activated RIPK1 has been shown to contribute to PANoptosome formation, and future studies are needed to deeply elucidate the underlying mechanistic roles of other PANoptosome sensor proteins (ZBP-1, AIM2, and NLRP12) in PANoptosome assembly and PANoptosis during *P. aeruginosa* infection.

##### Upregulation and/or Activation of Key Upstream Regulators of PANoptosome Complexes Upon *P. aeruginosa* Infection

As previously summarized, IRF1 is a key upstream regulator in PANoptosome assembly and PANoptosis. Interestingly, increased expression of IRF1 is observed in bladder epithelial cells upon *P. aeruginosa* infection [51]. Moreover, *P. aeruginosa* infection results in the upregulation of IRF1 in alveolar macrophages and lung tissue [52]. Given the pivotal role of IRF1 in PANoptosome assembly, these important findings suggest a connection between *P. aeruginosa*-induced IRF1 upregulation and PANoptosis. In another study, the N-(3-oxododecanoyl) homoserine lactone (3-oxo-C12-HSL) derived from *P. aeruginosa* causes caspase-8 activation [53], which plays a core role in mediating PANoptosis. Taken together, all the aforementioned findings clearly demonstrate that *P. aeruginosa* infection triggers the upregulation and/or activation of key upstream regulators and sensor proteins of PANoptosome complexes.

#### 2.1.4. Outstanding Questions in *P*. *aeruginosa*-Induced PANoptosis

Although prior studies have demonstrated that *P. aeruginosa* triggers PANoptosis and activates PANoptosome sensors and upstream regulators, precise functions of individual PANoptosome components are not well understood. Their mechanistic roles in PANoptosome assembly and PANoptosis should be studied in conditional knockout conditions, and their direct interplay should be revealed through high-resolution structures of PANoptosome complexes.

As previously mentioned, the QS system of *P. aeruginosa* is mechanistically involved in the occurrence of PANoptosis. As the *P. aeruginosa* QS system controls the production and activation of diverse virulence factors [54], such as toxins, pyocyanin, elastase, and proteases, future studies need to thoroughly investigate whether and how each of these virulence factor contributes to the emergence of PANoptosis, which has been extensively demonstrated in other well-studied PCD pathways. A special focus should be given to the virulence factors that have been proven to simultaneously induce several types of PCD, including apoptosis, pyroptosis, and necroptosis.

Additionally, different strains of *P. aeruginosa* may vary in their capacity to induce PANoptosis. In the aforementioned studies, researchers investigated *P. aeruginosa*-induced PANoptosis using the PAO1 strain. Although it is commonly used in the research, the PAO1 strain and clinical *P. aeruginosa* isolates may differ in their QS systems and virulence factors. Accordingly, to discover potential targeted therapeutic strategies against *P. aeruginosa* infection in clinics, there is an urgent need to thoroughly explore the precise roles and mechanisms of clinical *P. aeruginosa* isolates, particularly the multidrug-resistant (MDR) isolates, in PANoptosome assembly and PANoptosis.

Furthermore, despite confirming that *P. aeruginosa* triggers PANoptosis in macrophages, it still remains unknown whether *P. aeruginosa* triggers PANoptosis in other immune cells that play crucial roles in pathogen clearance (e.g., neutrophils and T cells). Given the strong cytotoxicity and pathogenicity of *P. aeruginosa*, this pathogen might induce PANoptosis in both innate and adaptive pathogen-clearing cells, thereby impeding both innate and adaptive immunity, a direction that strongly requires exploration.

### 2.2. P. aeruginosa-Induced Apoptosis

Apoptosis is characterized by nuclear fragmentation, chromatin condensation, DNA fragmentation, cellular shrinking and blebbing, and apoptosome formation [55,56]. Apoptosis is a fundamental biological process essential for maintaining hemostasis, and it also plays a vital role in regulating host immune defense in response to pathogen invasion [57]. At the molecular level, apoptosis is driven by apoptotic initiator caspases such as caspase-8, -9, and -10 [58,59]. Dimerization of initiator caspases facilitates their activation, and they subsequently promote the cleavage and activation of apoptotic effector caspases (e.g., caspase-3 and -7), which carry out the execution of apoptosis [58].

**Figure 3:**
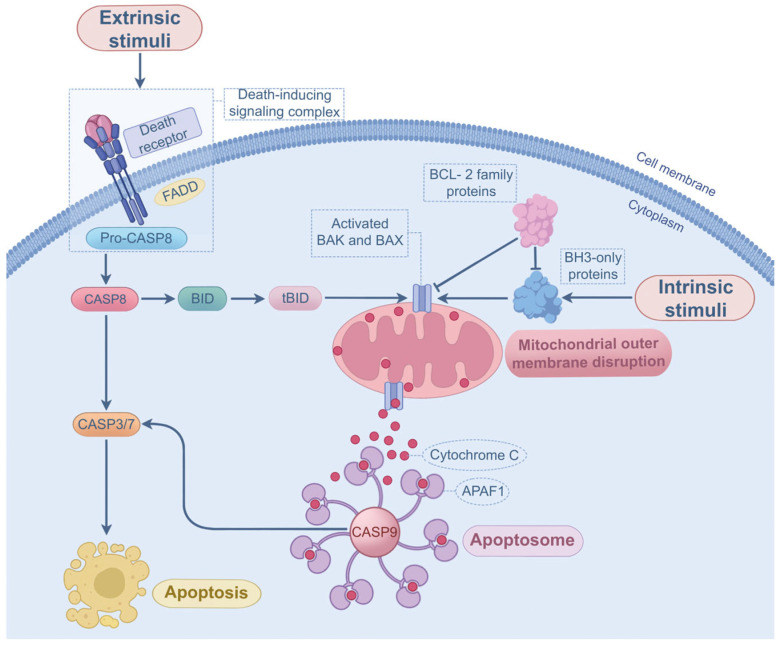
Intrinsic and extrinsic mechanisms of apoptosis. In intrinsic apoptosis, activation of BH3-only proteins facilitates the formation and oligomerization of BAK and BAX, leading to the release of cytochrome *c*, apoptosome assembly, and apoptosis. In extrinsic apoptosis, extracellular stimuli activate the death receptors, contributing to death-inducing signaling complex formation, procaspase-8 activation, and effector caspase activation.

Apoptosis can be classified into intrinsic and extrinsic pathways. In intrinsic apoptosis, BH3-only proteins (e.g., PUMA) are overexpressed and activated by diverse factors such as hypoxia and DNA damage [60]. When the activation of BH3-only proteins exceeds a specific threshold, the suppressive effect of anti-apoptotic B-cell lymphoma-2 (BCL-2) proteins (e.g., MCL1 and BCL-2) is counteracted, promoting the assembly and oligomerization of BAK and BAX in the outer membranes of mitochondria [61,62]. This allows cytochrome *c* to be released from the mitochondria into cytosol, where it interacts with the apoptotic initiator caspase-9 and apoptotic protease-activating factor-1 [63], ultimately contributing to apoptosome formation and apoptosis. The extrinsic pathway occurs upon ligands (extracellular stimuli) binding to death receptors (DRs) on the cell membrane. The DRs belong to the tumor necrosis factor (TNF) superfamily, such as TNFR1, Fas, DR4, and DR5, and their ligands are TNF-α, FasL, and TRAIL, respectively [64]. Activation of DRs is able to recruit the adaptor proteins (e.g., FADD), and the DRs, FADD, and procaspase-8 assemble into the death-inducing signaling complex, which facilitates the activation of procaspase-8 [62]. Once activated, caspase-8 provokes apoptosis by activating the effector caspases [62]. In some conditions, active caspase-8 is capable of cleaving Bid (a member of BH3-only proteins) into tBId [65], enabling the interaction between extrinsic and intrinsic apoptotic signaling. Apart from this, caspase-8 is a crucial molecular switch orchestrating the interplay between apoptosis and other PCD pathways such as necroptosis and pyroptosis [44]. The molecular mechanisms of apoptosis are shown in Figure 3.

#### Progress and Outstanding Questions in *P. aeruginosa*-Induced Apoptosis

Prior research has provided evidence that *P. aeruginosa* can induce apoptosis through various virulence factors. For instance, T3SS-secreted ExoS leads to intrinsic apoptosis via activation of the apoptotic initiator caspase-9 and effector caspase-3 [15], and the FADD/caspase-8-dependent extrinsic pathway is also found in response to ExoS treatment [66]. The GTPase-activating protein domain of T3SS-secreted ExoT exhibits the ability to induce caspase-9-dependent intrinsic apoptosis [67]. Moreover, characteristic features of apoptosis are observed following *P. aeruginosa* ExoY exposure [68]. Aside from the T3SS-secreted toxins, the porin from *P. aeruginosa* is reported to initiate the apoptotic pathway through inhibiting BCL-2 expression [69]. Similarly, LPS, another virulence factor of *P. aeruginosa*, is recognized for its ability to drive apoptosis [70]. Pyocyanin, a potent cytotoxic pigment produced by *P. aeruginosa*, contributes to mitochondrial injury and cytochrome *c* release [71], suggesting induction of the intrinsic apoptotic pathway. Furthermore, the 3-oxo-C12-HSL derived from *P. aeruginosa* drives spontaneous trimerization of TNFR1, promoting caspase-8 and -3-mediated extrinsic apoptosis [72]. Intrinsic apoptosis is also detected after 3-oxo-C12-HSL exposure, but is completely abolished by BCL-2 overexpression [73]. In addition to the virulence factors discussed above, the bacterial ExoA mediates intrinsic apoptosis since ExoA treatment causes MCL1 degradation and BAK activation [74]. Besides that, ExoA facilitates extrinsic apoptosis via activating caspase-8 and -3 [75]. It is notable that pyocyanin-induced neutrophil apoptosis impairs host bacterial clearance and inflammatory response [76]. Nevertheless, results from another study reveal that *P. aeruginosa*-induced apoptosis is important for lung bacterial clearance, and defective apoptosis in human bronchial epithelial cells is likely to contribute to the pathogenesis of persistent *P. aeruginosa* colonization in cystic fibrosis [77]. Based on these aforementioned findings, it is important to note that, although *P. aeruginosa* is well known to trigger apoptosis, it still remains controversial whether *P. aeruginosa*-induced apoptosis is beneficial or detrimental for bacterial eradication and infection resolution. In our perspective, underlying mechanisms of this question may be complex and multifaceted. Future studies should thoroughly investigate this question by comprehensively considering factors such as strain diversity (e.g., differences in virulence factor expression between strains), organ and tissue specificity, target cells, and complex bacteria-host interactions.

### 2.3. P. aeruginosa-Induced Pyroptosis

**Figure 4:**
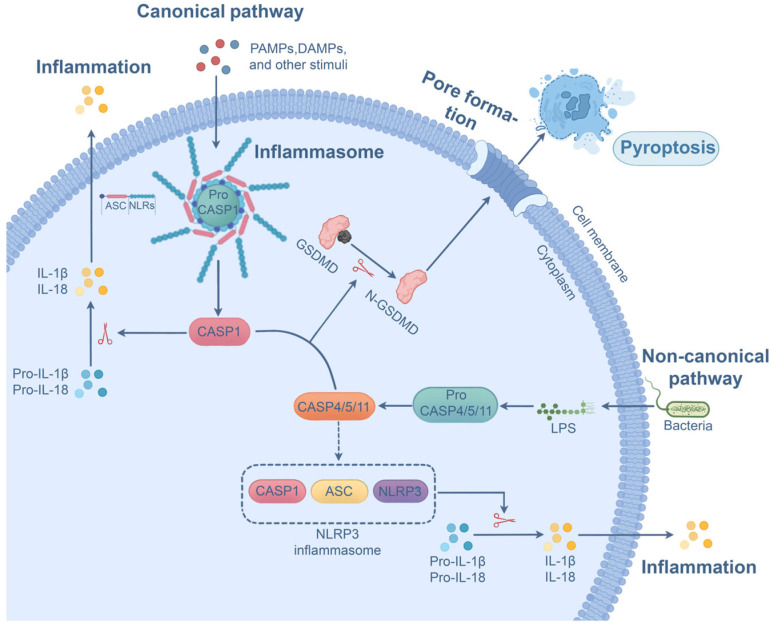
Canonical and non-canonical pyroptosis pathways. In canonical pyroptosis, PAMPs and DAMPs promote inflammasome formation and caspase-1 activation. Activated caspase-1 cleaves GSDMD into N-GSDMD, perforating the plasma membrane and causing pyroptosis. In the non-canonical pathway, caspase-4, -5, and -11 undergo activation through binding with LPS, and these caspases subsequently cleave GSDMD for pore formation and pyroptosis. Meanwhile, active caspase-1, as well as active caspase-4, -5, and -11, contribute to the maturation of IL-1β and IL-18 directly or indirectly.

Contrary to apoptosis, pyroptosis is a highly pro-inflammatory PCD pathway with distinct morphological changes such as plasma membrane rupture, membrane blebbing, and cytoplasm flattening [78]. Pyroptosis can be induced by either the canonical or non-canonical pathway. The canonical pyroptotic pathway is mediated by inflammasomes (e.g., NLRP3 inflammasome or NLRC4 inflammasome), a multiprotein complex comprising cytoplasmic sensor NOD-like receptors (NLRs) or AIM2/Pyrin, procaspase-1, and the adaptor ASC [79]. The assembly of inflammasomes is initiated upon detection of PAMPs and danger-associated molecular patterns (DAMPs) by NLRs [79,80]. The oligomerization and activation of NLRs engage ASC, an adaptor bridging the sensors (NLRs or AIM2/Pyrin) and the procaspase-1 [80]. Procaspase-1 is subsequently recruited, followed by self-cleavage and activation [81]. The activated form of caspase-1 mediates the cleavage of GSDMD and the generation of N-terminal domain (N-GSDMD), forming pores on the cell membrane and promoting pyroptosis [82]. Meanwhile, active caspase-1 drives IL-1β and IL-18 maturation through cleavage of their inactive precursors (proIL-1β and proIL-18), after which they are released through pores created by N-GSDMD [79,83]. In non-canonical pyroptosis, upon direct binding to LPS, caspase-4, -5, and -11 undergo oligomerization and activation [84]. Active forms of these caspases cleave GSDMD to produce N-GSDMD, resulting in plasma membrane pore formation and pyroptosis. In addition, these caspases indirectly contribute to the maturation of IL-18 and IL-1β in an NLRP3 inflammasome-dependent manner in certain cells [85]. It is notable that other Gasdermin family proteins, such as Gasdermin B and GSDME, are found to execute pyroptosis through their pore-forming activity after being cleaved [40,86]. Figure 4 depicts the molecular pathways of pyroptosis.

#### Progress and Outstanding Questions in *P. aeruginosa*-Induced Pyroptosis

Previously published studies have provided evidence that diverse virulence factors of *P. aeruginosa* can cause pyroptosis. For instance, flagellin, a pathogenic protein from *P. aeruginosa*, causes pyroptotic cell death through NLRC4 inflammasome activation [87,88]. In the presence of ExoS ADP ribosyl transferase, neutrophils undergo pyroptosis in an NLRP3-dependent manner [89]. Intriguingly, caspase-11-dependent non-canonical pyroptosis also occurs during T3SS-negative *P. aeruginosa* infection [90]. The exolysin secreted by *P. aeruginosa* was identified to provoke pyroptotic cell death through activation of the NLRP3 inflammasome and downstream caspase-1 [17]. It is noteworthy that, contrary to its apoptosis-inducing capacity, pyocyanin suppresses inflammasome activation and caspase-1-dependent canonical pyroptosis, which enables bacteria to evade immune surveillance [91]. Aside from the aforementioned virulence factors, deficiency in oprC (one of the *P. aeruginosa* porins) reduces GSDMD cleavage and IL-1β release, implicating the role of oprC in pyroptosis induction [92]. Augmented levels of active caspase-1, IL-18, and IL-1β are also detected following *P. aeruginosa* biofilm stimulation [93], indicating that the bacterial biofilm mediates pyroptotic cell death. The pyroptosis induced by bacterial virulence factors is usually considered a host defense mechanism to restrict infection through activation of the pro-inflammatory immune response and, ultimately, sacrifice of infected cells. In line with this, in an animal model of *P. aeruginosa*-induced keratitis, mice deficient in caspase-1 and caspase-1/11 exhibit higher bacterial load and more severe corneal disease [89]. Nevertheless, NLRC4 inflammasome activation and the resulting IL-18 generation are reported to suppress host antimicrobial response against the *P. aeruginosa* strain with functional T3SS, leading to reduced bacterial clearance and an increased level of lung injury [94]. Similarly, findings from Cohen and colleagues unveil that inhibition of caspase-1 and IL-18/IL-1β improves the clearance of *P. aeruginosa* and alleviates the severity of *P. aeruginosa* pulmonary infection [95]. Accordingly, these findings suggest that the precise role of pyroptosis in host defense to *P. aeruginosa* invasion is not well determined. Given the variations in virulence factor expression among different strains, the pathophysiological effect of *P. aeruginosa*-induced pyroptosis may significantly vary. Importantly, when a *P. aeruginosa* strain harbors both pyroptosis-inducing (e.g., T3SS) and pyroptosis-inhibiting (e.g., pyocyanin) virulence factors, their multifaceted impact on pyroptosis remains uncertain. Furthermore, the complexity of bacteria-host interactions may be another determinant for *P. aeruginosa*-induced pyroptosis. For example, different organs and tissues exhibit a distinct microenvironment and composition of resident cells. Consequently, the target cells undergoing *P. aeruginosa*-induced pyroptosis may differ, and the role of pyroptosis in bacterial eradication and disease resolution may alter.

### 2.4. P. aeruginosa-Induced Necroptosis

**Figure 5:**
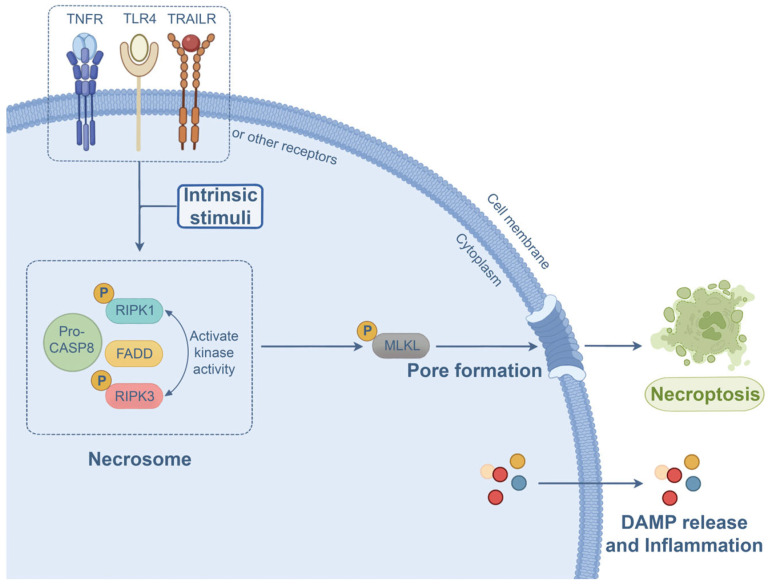
Necroptosis signaling pathway. Activation of multiple receptors or intrinsic stimuli causes RIPK1 and RIPK3 phosphorylation and necrosome assembly (phosphorylated RIPK1 and RIPK3, FADD, and inactive caspase-8). Thereafter, MLKL undergoes phosphorylation and migrates to the plasma membrane, where it oligomerizes to drive pore formation and necroptosis. In the meantime, cell contents and DAMPs are released through pores to provoke inflammation.

Necroptosis is a form of pro-inflammatory PCD, and it is morphologically characterized by cellular and organelle edema, cell membrane damage, and cell content release [78,96]. Necroptosis acts as a complementary pathway for cell death if caspase-8-mediated apoptosis is disrupted [97]. Necroptosis is initiated following the activation of TNFRs [98,99,100], TLR3/4 [101,102], Fas [98,103], TRAILR [104], and NLR [105], as well as upon stimulation by various factors such as IFNs [106], hypoxia [107], and bacterial infection [108]. Subsequently, the interaction and phosphorylation of RIPK1 and RIPK3 activate pronecrotic kinase activity [109,110] and promote the formation of the necroptosis signaling complex called the necrosome [111]. Activated RIPK3 facilitates the recruitment and phosphorylation of the necroptosis effector MLKL [38,112]. After phosphorylation, MLKL undergoes translocation and oligomerization in the plasma membrane [113], perforating the membrane to form necroptotic pores. In the meantime, necroptotic cells release cell contents and DAMPs that elicit substantial inflammatory responses through permeabilized membranes [114]. Besides RIPK1, RIPK3 can also interact with either TRIF or ZBP-1 for the induction of necroptosis [115,116]. The necroptosis signaling pathway is illustrated in Figure 5.

#### Progress and Outstanding Questions in *P. aeruginosa*-Induced Necroptosis

It has been confirmed that *P. aeruginosa* virulence factors are critically involved in the process of necroptosis. The exolysin secreted by *P. aeruginosa* was demonstrated to mediate necroptosis as the use of RIPK1 and RIPK3 inhibitors diminishes exolysin-induced cytotoxicity [17]. The bacterial QS system also plays a role in regulating host necroptosis during infection [22]. *P. aeruginosa* LPS interrupts the binding of RIPK3 to histone and facilitates subsequent RIPK3 translocation [117], implying that LPS is involved in the regulation of necroptosis signaling. In contrast to apoptosis and pyroptosis, pyocyanin is not involved in necroptosis signaling, because pyocyanin-induced cytotoxicity is not reversed by the necroptosis inhibitor [118]. It is worth mentioning that a prior study unveils that *P. aeruginosa*-mediated necroptosis contributes to disease pathology as the pharmacological inhibition of necroptosis signaling mitigates the severity of acute lung injury [119]. Conversely, results from another study show that the necroptosis initiator RIPK3 functions as an attenuator of pulmonary inflammation caused by *P. aeruginosa* after its translocation from the nucleus to the cytoplasm, although the activation of necroptosis signaling was not assessed in this research [117]. Thus, similar to aforementioned PCD pathways, the aforementioned findings highlight the need to clarify whether *P. aeruginosa*-induced necroptosis is host-beneficial or -detrimental. This question should be explored through comprehensive consideration of multiple factors such as strain diversity, organ and tissue specificity, and host immune status.

### 2.5. P. aeruginosa-Induced Ferroptosis

**Figure 6:**
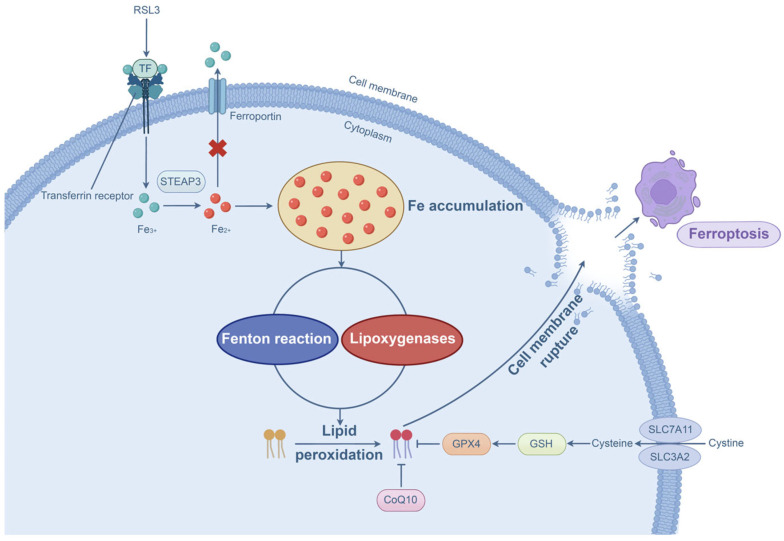
Molecular processes of ferroptosis. Iron accumulation (e.g., RSL3, increased transferrin expression, and declined ferroportin expression) and lipid peroxidation are two major contributors to ferroptosis. Lipid peroxidation occurs via the non-enzymatic (Fenton reaction) or lipoxygenase-mediated enzymatic pathway, resulting in membrane rupture and ferroptosis. Inhibition of GPX4 or CoQ10 also elicits ferroptosis.

Ferroptosis is a form of PCD with distinct morphological features such as shrunken mitochondria, loss of mitochondrial cristae, and disruption of outer mitochondrial membrane integrity [120]. Notably, unlike other types of PCD described earlier, a normal nuclear size, lack of chromatin condensation, and intact plasma membrane are observed in cells undergoing ferroptosis [120]. Ferroptosis primarily occurs through iron accumulation and lipid peroxidation. The ferroptosis inducer RSL3 can induce intracellular iron accumulation [121]. Moreover, enhanced transferrin expression and declined ferroportin expression lead to iron accumulation [122]. Accumulated Fe^3+^ can be converted into Fe^2+^ by the STEAP3 metalloreductase, resulting in the excessive production of reactive oxygen species through the Fenton reaction [123]. More importantly, this reaction mediates the non-enzymatic peroxidation of polyunsaturated fatty acids [124,125], a key pathway for ferroptosis initiation. Compared with the non-enzymatic pathway, enzymatic lipid peroxidation is controlled by lipoxygenases, which modulate the peroxidation of polyunsaturated fatty acids for ferroptosis induction [126,127]. Furthermore, inactivation and depletion of GPX4 and its crucial cofactor GSH elicit ferroptotic cell death because GPX4 is responsible for detoxification of the hydroperoxides [126]. In addition to GPX4, the coenzyme Q10 functions as another important antioxidant that halts lipid peroxidation [128]. Figure 6 presents the molecular processes of ferroptosis.

#### Progress in *P. aeruginosa*-Induced Ferroptosis

It has been evidenced that bacterial infections trigger ferroptosis [129], including Gram-negative bacteria [130,131]. Among them, *P. aeruginosa* was demonstrated to generate lipoxygenase (pLoxA), which promotes host lipid peroxidation (from arachidonic acid-phosphatidylethanolamines to 15-hydroperoxy-arachidonic acid-phosphatidylethanolamines) and ferroptosis [132]. Another study reveals that *P. aeruginosa* is able to degrade GPX4 activity [133]. Similarly, inhibition of the anti-ferroptotic pathway (GSH/GPX4) and activation of the pro-ferroptotic signal have been found during *P. aeruginosa* infection, and the use of the *P. aeruginosa* lipoxygenase inhibitor mitigates the ferroptotic signal and disease severity [134]. Importantly, a previous study discovered that ferroptosis inhibitor liproxstatin-1 is capable of blocking PANoptosis by suppressing the cleavage of PANoptosome upstream regulators (caspase-8 and -6) [135]. Results from another study indicate that there exists an association and cross-talk between ferroptosis and PANoptosis under hypoxic conditions [136]. Given that the proliferation and pathogenicity of *P. aeruginosa* are closely associated with anaerobic conditions [137], new treatment strategies for *P. aeruginosa*-induced PANoptosis, particularly caused by MDR strains, warrant further exploration by considering the interplay between ferroptosis and PANoptosis as well as the potential use of ferroptosis inhibitors to target PANoptosis.

## 3. Potential Targeted Treatment Strategies for *P. aeruginosa*-Induced PCD

*P. aeruginosa* infection poses a serious risk to patients due to its antibiotic resistance capacity. As one of the major pathogenic mechanisms of *P. aeruginosa* is to induce multiple forms of PCD, targeting them may provide alternative therapeutic options for resolving *P. aeruginosa* infection, particularly for infection caused by MDR strains. As potential treatments for well-studied PCD pathways (e.g., apoptosis, pyroptosis, and necroptosis) have been discussed by previous studies, we herein focus on discussing pharmacological inhibitors and compounds targeting the recently discovered *P. aeruginosa*-induced PANoptosis.

### 3.1. Targeting Key Upstream Regulators

As previously discussed, *P. aeruginosa* triggers PANoptosis, and previous studies have confirmed that *P. aeruginosa* infection results in the upregulation of IRF1. Intriguingly, genetic depletion or pharmacological inhibition of IRF1 attenuates IRF1-mediated inflammatory cell death [19,138] and reduces the cleavage of caspase-1 and GSDMD [138]. These results suggest that targeted disruption of IRF1 using specific inhibitors (e.g., I-2 and I-19) may be a potential therapeutic approach for *P. aeruginosa*-induced PANoptosis. Importantly, no obvious multiorgan side effects have been observed in mice treated with I-2 or I-19 [138], indicating that they may be suitable for clinical translation after human trials. In a prior study, treatment with necrostatin-1 (an RIPK1 inhibitor) reverses the activation of caspase-8 and alleviates the cell death resulting from stimulation of *P. aeruginosa*-secreted 3-oxo-C12-HSL [53]. Another study reveals that miR-29a-3p exhibits the capacity of suppressing caspase-8 activation and PANoptosis in alveolar epithelial cells [139]. Furthermore, administration of the caspase-8 inhibitor Z-IETD-FMK enhances neutrophil antimicrobial activity in the absence of increased cell death [140]. These findings indicate that blockade of caspase-8 activation using pharmacological compounds or small molecules offers a possible treatment strategy for *P. aeruginosa*-induced PANoptosis. Another investigation shows that caspase-8 activation occurs downstream of caspase-6, another core upstream regulator of PANoptosis, and the use of a selective caspase-6 inhibitor Z-VEID-FMK prevents apoptotic cell death and facilitates cell survival [141]. This underscores the important role of caspase-6 in controlling cell death, implicating the therapeutic potential of applying caspase-6 inhibitors against *P. aeruginosa*-induced PANoptosis. Nevertheless, necrostatin-1 and the aforementioned caspase inhibitors have not yet entered clinical trials, and their efficacy and safety for the treatment of *P. aeruginosa*-induced PANoptosis still need further investigation and optimization.

### 3.2. Targeting Sensor Proteins

As discussed earlier, ZBP-1 is a pivotal sensor protein that composes the PANoptosome and modulates the process of PANoptosis. It is reported that ADAR1 can limit the activation of ZBP-1, preventing ZBP-1-mediated cell death and autoinflammation [142]. ADAR1 has been involved in clinical investigations in the field of cancer therapy efficacy (NCT07108348 and NCT05482451), and its clinical feasibility in treating *P. aeruginosa*-induced PANoptosis warrants further assessment. The miR-29a-3p treatment also diminishes the levels of ZBP-1 and PANoptosis [139]. Furthermore, a prior study shows that CDK1 can bind with ZBP-1 PANoptosome, and the administration of cucurbitacin E (a CDK1 inhibitor) is able to limit tumor cell growth by regulating PANoptosis [143]. With regard to other PANoptosome sensor proteins, IFI16-β represses AIM2 activation since IFI16-β can prevent the AIM2 recognition of cytosolic dsDNA [144]. In addition, IFI16-β impairs the assembly of AIM2-ASC complex [144]. J114 also displays a potent inhibitory effect on AIM2-dependent cell death as it disturbs AIM2-ASC interaction and dampens AIM2 activation [145]. 4-Sulfonic calix[6]arene was identified as another AIM2 inhibitor because it blocks the DNA-binding HIN domain of AIM2 [146]. In this study, researchers have confirmed that suramin, a clinically available polysulfonated drug exhibiting similar properties to 4-Sulfonic calix[6]arene, effectively inhibits AIM2 inflammasome activation [146], suggesting the feasibility for clinical use. In comparison with ZBP-1 and AIM2, many potent and highly specific RIPK1 inhibitors have been discovered over the past decade. 6E11, a highly selective human RIPK1 inhibitor, is shown to restrict TNF-α (one of the PANoptosis inducers [19])-induced, RIPK1-driven cell death while exhibiting low cytotoxicity toward normal cells [147]. This high-affinity, non-ATP competitive inhibitor provides more potent protection for cell survival compared with necrostatin-1, a well-studied RIPK1 inhibitor [147]. Further animal and clinical studies are needed to evaluate its clinical potential. ZB-R-55 was found to be another highly effective RIPK1 inhibitor with desirable kinase selectivity and therapeutic effects in sepsis induced by LPS (a major virulence factor of *P. aeruginosa*) [148]. Importantly, ZB-R-55 shows approximately tenfold greater potency compared with GSK2982772 [148], the first RIPK1 inhibitor that entered clinical investigations. Moreover, treatment with RIPA-56 efficiently protects mice from TNF-α-induced mortality, systemic inflammation, and multiorgan damage, proving its high effectiveness, selectivity, and metabolic stability for RIPK1 inhibition [149]. Given the high selectivity, efficacy, and metabolic stability of these specific RIPK1 inhibitors in preclinical models, their therapeutic potential and adverse effects require further investigation in clinical settings. As for NLRP12, there are currently no direct NLRP12 inhibitors available for human or animal studies. Screening, optimization, and application of NLRP12 inhibitors are strongly encouraged to evaluate their specificity and potency in limiting NLRP12-mediated PCD, especially in the context of *P. aeruginosa* infection.

### 3.3. Targeting Other Molecular Components of PANoptosome Complexes or Downstream Executioners

ASC, an important adaptor protein bridging the upstream signaling and downstream effectors for PANoptosome assembly and activation, can be regarded as a potential therapeutic target. Spirodalesol analog 8A was confirmed to be a desirable ASC inhibitor as it directly interacts with ASC to prevent ASC speck formation [150]. Treatment of 8A also attenuates the severity of endotoxemia induced by LPS [150]. As previously reviewed, pharmacological inhibition of AIM2-ASC interaction and ASC oligomerization by J114 protects cells from AIM2-dependent cell death [145]. Since AIM2 and ASC play pivotal roles in modulating PANoptosis, the potential of applying these selective ASC inhibitors against *P. aeruginosa*-induced PANoptosis is promising. Besides ASC, caspase-1 is another important component of the PANoptosome. Tetracycline is evidenced to mitigate caspase-1-dependent cell death and lung inflammation [151]. Notably, tetracycline dose-dependently reduces IL-18 and IL-1β production by alveolar leukocytes from patients with acute lung inflammation [151]. This finding strengthens the feasibility of applying tetracycline to target caspase-1 in clinical settings. The unsaturated ester derivative-compound 9 blocks caspase-1 activity and prevents caspase-1-driven cell death in a time- and concentration-dependent manner [152]. However, although compound 9 shows strong protective efficacy against caspase-1-driven cell death, its high cytotoxicity needs further evaluation in animal studies. A prior study reports that compound 42 (an analog of TAK-632) attenuates RIPK3-dependent cell death through inhibiting the phosphorylation of RIPK3 [153], another cell death mediator. Furthermore, compound 42 exhibits over 60-fold selectivity for RIPK3 over RIPK1 [153]. AZD5423 can also directly target RIPK3 to repress its kinase activity, impeding RIPK3 activation and alleviating RIPK3-dependent cell damage [154]. It is notable that these RIPK3 inhibitors are not yet clinically available. However, as an important component of PANoptosome complexes, RIPK3 still remains a promising therapeutic target for *P. aeruginosa*-induced PANoptosis.

Aside from the PANoptosome components described before, GSDMD and MLKL (downstream executioners) may be targetable molecules for mitigating cell death. For instance, necrosulfonamide (NSA) is able to reduce GSDMD-mediated cell death and ameliorate LPS-induced sepsis through directly binding to the Cys191 of GSDMD [155]. Through the blockade of GSDMD’s pore-forming activity, disulfiram exerts an inhibitory effect on pyroptotic cell death and LPS-induced sepsis in mice [156]. As mentioned earlier, LPS serves as a major pathogenic factor of *P. aeruginosa*, implicating the possibility of employing NSA or disulfiram against *P. aeruginosa*-induced PANoptosis. Surprisingly, cell death triggered by the AIM2 inflammasome is also impaired by disulfiram in a dose-dependent manner [156], suggesting that disulfiram can target both AIM2 and GSDMD. As an FDA-approved anti-alcoholism agent, this dual-target drug shows great promise against *P. aeruginosa*-induced PANoptosis, provided that its dosage, pharmacodynamics, and oral bioavailability are carefully assessed in clinical trials. With regard to MLKL, treatment with NSA can downregulate its phosphorylation and activation [157]. Mechanistically, the Cys86 of human MLKL was identified as the covalent binding and modification site for NSA [38]. Thus, NSA acts as a dual-target inhibitor of GSDMD and MLKL. Nevertheless, unlike disulfiram, NSA is not yet available for clinical use, and thus clinical investigations are needed to study the efficacy and safety of this promising dual-target drug. Saracatinib, a drug with encouraging preclinical and clinical results for multiple diseases [158], suppresses TNF-induced cell death by disrupting phosphorylation, translocation, and oligomerization of MLKL [159]. All aforementioned pharmacological compounds and small molecules are summarized in Table 1. Recently, it has been reported that Imipenem-Relebactam treatment shows a high clinical success rate (75.9%) and reduced 30-day mortality (24.1%) in patients with difficult-to-treat *P. aeruginosa* infection [160]. This highlights the potential of combining the aforementioned pharmacological compounds or small molecules with antibiotics to provide more favorable efficacy against *P. aeruginosa* infection.

## 4. Conclusions and Future Directions

*P. aeruginosa* is responsible for the onset of many hospital-acquired infections, and it still remains challenging to fully eradicate this pathogen in clinics. It has been demonstrated that it triggers multiple types of PCD, such as apoptosis, pyroptosis, necroptosis, ferroptosis, and PANoptosis, the latter of which is intricately regulated by PANoptosome complexes and their key upstream regulators. Progress in our understanding of these molecules, as well as their upregulation and/or activation in response to *P. aeruginosa* infection, offers up-to-date and comprehensive insights into the close mechanistic association between *P. aeruginosa* infection and PANoptosis. In addition to PANoptosis, *P. aeruginosa* virulence factors induce apoptosis, pyroptosis, necroptosis, and ferroptosis, and they are closely associated with the host’s immune reaction in response to *P. aeruginosa* invasion. Targeting *P. aeruginosa*-induced PCD using pharmacological compounds or small molecules may be an alternative treatment strategy for *P. aeruginosa* infection, especially for infection caused by MDR strains. Our efforts help bridge the knowledge gap and pave the way for future research to further understand the pathogenicity of *P. aeruginosa*.

However, although PANoptosome complexes and their key upstream regulators have been identified as PANoptosis mediators, little is known about the precise mechanistic role of each PANoptosome component in PANoptosis. To elucidate their functions and potential functional redundancies, conditional knockout experiments for each molecule in cells or animal models are needed. In vivo studies are preferable since host microenvironmental factors (e.g., pH and metabolites) and interactions among innate and adaptive immune cells may also influence the PANoptosis process. Furthermore, it is of great value to investigate how individual virulence factors of *P. aeruginosa* mechanistically contribute to PANoptosome assembly and PANoptosis. Besides laboratory *P. aeruginosa* strains, clinical MDR isolates should be used for research. To precisely define the cytotoxicity of a specific virulence factor, loss-of-function mutant strains can be generated and utilized. Moreover, it is crucial to clarify the precise roles of *P. aeruginosa*-induced PCD and identify when it plays a beneficial role (e.g., restricting infection or activating immune response) or detrimental role (e.g., mediating organ and tissue damage) at different phases of infection. Lastly, the development of clinically effective medications targeting *P. aeruginosa*-induced PCD is highly anticipated. Considering recent advances in antibiotic treatment against *P. aeruginosa* infection, combining *P. aeruginosa*-induced PCD-targeting agents with antibiotics may achieve more favorable efficacy for *P. aeruginosa* infection. Unveiling the high-resolution structures of PANoptosome complexes is of great significance, as it will substantially facilitate structure-based drug design. Thereafter, the translational feasibility of drugs, such as selectivity, metabolic stability, and oral bioavailability, can be further evaluated and optimized in preclinical models. Finally, clinical studies are indispensable for validating therapeutic effectiveness and assessing potential side effects. Taken together, gaining new knowledge and insights into *P. aeruginosa*-induced PCD will open new avenues for developing effective targeted therapeutics against *P. aeruginosa* infection.

## Figures and Tables

**Table 1:** Potential pharmacological compounds and small molecules targeting *P. aeruginosa*-induced PANoptosis.

Compound/ Small Molecule	Target and Mechanism	Disease/Condition	Outcome	Refs
I-2	IRF1 Forms hydrogen bonds and hydrophobic contacts with IRF; reduces IRF1 transcriptional activity	Radiation-induced inflammatory cell death and tissue injury	Inhibits cleavage of PANoptotic molecules and mitigates cell death and tissue injury	[138]
I-19	IRF1 Targets DNA-binding domain of IRF1; reduces IRF1 transcriptional activity	Radiation-induced inflammatory cell death and tissue injury	Inhibits cleavage of PANoptotic molecules and mitigates cell death and tissue injury	[138]
Necrostatin-1	caspase-8 Decreases caspase-8 cleavage	Cell death induced by *P. aeruginosa*-derived 3-oxo-C12-HSL	Suppresses cell death in 3-oxo-C12-HSL-treated cells	[53]
miR-29a-3p	caspase-8 and ZBP-1 Inhibits caspase-8 activation and ZBP-1 expression	Acute lung injury	Reduces alveolar epithelial cell PANoptosis and alleviates disease severity	[139]
Z-IETD-FMK	caspase-8 Inhibits caspase-8 cleavage	Bacterial peritonitis, pneumonia, and endotoxin shock	Enhances neutrophil antimicrobial activity in the absence of increased cell death	[140]
Z-VEID-FMK	caspase-6 Selectively and irreversibly inhibits caspase-6	Optic nerve injury	Reduces apoptotic cell death and promotes cell survival	[141]
ADAR1	ZBP-1 Zα domain of ADAR1 limits ZBP1 activation	Embryonic lethality, intestinal cell death, and skin inflammation	Prevents ZBP1-induced cell death and autoinflammation	[142]
Cucurbitacin E	CDK1/ZBP-1 Inhibits CDK1, a protein mediating PANoptosis via interaction with ZBP-1 PANoptosome	Adrenocortical carcinoma	Regulates PANoptosis in cancer cells	[143]
IFI16-β	AIM2 Impairs AIM2-ASC interaction and AIM2-dsDNA sensing	Bacterial and viral infection	Suppresses AIM2 inflammasome activation and pro-inflammatory cytokine release	[144]
J114	AIM2 Disturbs AIM2-ASC interaction	AIM2-dependent inflammation	Reduces PANoptotic molecule expression and pro-inflammatory cytokine release	[145]
4-Sulfonic calix[6]arene/suramin	AIM2 Blocks DNA-binding HIN domain of AIM2	Post-stroke immunosuppression	Prevents AIM2 activation and AIM2-dependent cell death	[146]
6E11	RIPK1 Competes with RIPK1-ligand complexes	TNF-α-induced cell death and cold hypoxia/reoxygenation injury	Protects against RIPK1-dependent cell death	[147]
ZB-R-55	RIPK1 Occupies allosteric and ATP binding pockets of RIPK1	Sepsis and systemic inflammatory response syndrome	Attenuates systemic inflammation and disease severity	[148]
RIPA-56	RIPK1 Inhibits RIPK1 phosphorylation and its kinase activity	Systemic inflammatory response syndrome	Prevents multiorgan damage and reduces mortality	[149]
Spirodalesol analog 8A	ASC Suppresses ASC speck formation and oligomerization	Endotoxemia, peritonitis, and gouty arthritis	Reduces mortality and multiorgan damage	[150]
Tetracycline	caspase-1 Reduces cleavage and activation of caspase-1	Acute lung injury	Attenuates lung injury and pulmonary inflammation; improves survival	[151]
Unsaturated ester derivative-compound 9	caspase-1 Blocks caspase-1 activity	Caspase-1-dependent cell death	Protects against caspase-1-dependent cell death	[152]
Compound 42 (an analog of TAK-632)	RIPK3 Inhibits RIPK3 phosphorylation and blocks necrosome formation	Systemic inflammatory response syndrome	Alleviates disease symptoms and improves survival	[153]
AZD5423	RIPK3 Binds to the kinase domain of RIPK3 and decreases its activation	RIPK3-mediated cell injury and acute kidney injury	Restores cell viability and attenuates kidney injury and inflammation	[154]
Necrosulfonamide (NSA)	GSDMD Binds to the Cys191 of GSDMD MLKL Binds to the Cys86 of human MLKL; inhibits MLKL phosphorylation	LPS-induced endotoxicity and sepsis	Prevents cell death and reduces mortality	[155]
		Acute colitis	Alleviates intestinal inflammation	[38,157]
Disulfiram	GSDMD Modifies the Cys191 of human GSDMD AIM2 Mechanism unknown	Sepsis	Improves survival and attenuates systemic inflammation	[156]
Saracatinib	MLKL Disrupts the phosphorylation, translocation, and oligomerization of MLKL	Psoriasiform dermatitis	Attenuates skin inflammation	[159]

## Data Availability

No new data were created or analyzed in this study. Data sharing is not applicable to this article.
